# *QuickStats:* Deaths Involving Exposure to Excessive Heat,* by Sex — National Vital Statistics System, United States, 1999–2020

**DOI:** 10.15585/mmwr.mm7134a5

**Published:** 2022-08-26

**Figure:**
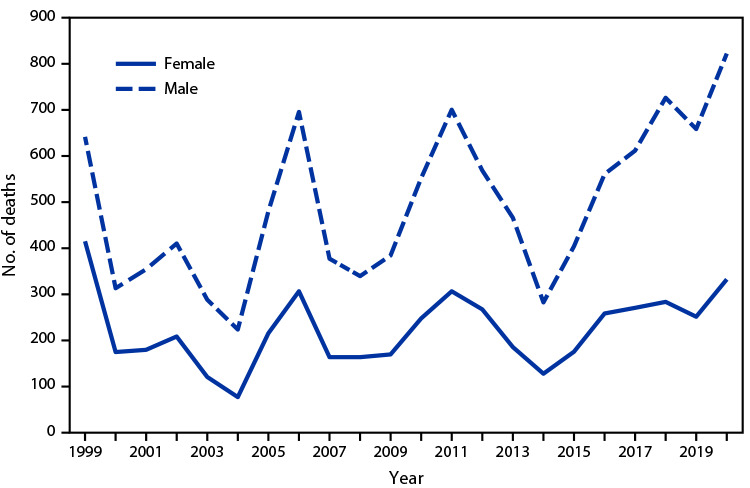
During 1999–2020, the annual number of deaths from excessive natural heat ranged from a low of 297 in 2004 to a high of 1,153 in 2020. The number of deaths among males increased from 622 deaths in 1999 to 822 deaths in 2020, but there was no statistically significant increase among females. During 1999–2020, there were generally twice as many deaths among males than among females each year.

**Source:** National Vital Statistics System, multiple cause of death data, 1999–2020. https://wonder.cdc.gov/mcd.html

For more information on this topic, CDC recommends the following link: https://www.cdc.gov/disasters/extremeheat/index.html

